# Alternative pathway to photorespiration protects growth and productivity at elevated temperatures in a model crop

**DOI:** 10.1111/pbi.13750

**Published:** 2021-11-24

**Authors:** Amanda P. Cavanagh, Paul F. South, Carl J. Bernacchi, Donald R. Ort

**Affiliations:** ^1^ Carl R. Woese Institute for Genomic Biology University of Illinois Urbana IL USA; ^2^ Global Change and Photosynthesis Research Unit United States Department of Agriculture–Agricultural Research Service Urbana IL USA; ^3^ Departments of Plant Biology and Crop Sciences University of Illinois Urbana IL USA; ^4^ Present address: School of Life Sciences University of Essex Colchester UK; ^5^ Present address: Department of Biological Sciences Louisiana State University Baton Rouge LA USA

**Keywords:** climate warming, photorespiration, engineering photosynthesis

## Abstract

Adapting crops to warmer growing season temperatures is a major challenge in mitigating the impacts of climate change on crop production. Warming temperatures drive greater evaporative demand and can directly interfere with both reproductive and vegetative physiological processes. Most of the world’s crop species have C3 photosynthetic metabolism for which increasing temperature means higher rates of photorespiration, wherein the enzyme responsible for fixing CO_2_ fixes O_2_ instead followed by an energetically costly recycling pathway that spans several cell compartments. In C3 crops like wheat, rice and soybean, photorespiration translates into large yield losses that are predicted to increase as global temperature warms. Engineering less energy‐intensive alternative photorespiratory pathways into crop chloroplasts drives increases in C3 biomass production under agricultural field conditions, but the efficacy of these pathways in mitigating the impact of warmer growing temperatures has not been tested. We grew tobacco plants expressing an alternative photorespiratory pathway under current and elevated temperatures (+5 °C) in agricultural field conditions. Engineered plants exhibited higher photosynthetic quantum efficiency under heated conditions than the control plants, and produced 26% (between 16% and 37%) more total biomass than WT plants under heated conditions, compared to 11% (between 5% and 17%) under ambient conditions. That is, engineered plants sustained 19% (between 11% and 21%) less yield loss under heated conditions compared to non‐engineered plants. These results support the theoretical predictions of temperature impacts on photorespiratory losses and provide insight toward the optimisation strategies required to help sustain or improve C3 crop yields in a warming climate.

## Introduction

It is projected that crop productivity must increase by 25%–70% over 2017 production levels to meet the anticipated agricultural demand in 2050 (Hunter *et al*., 2017). At the same time, agricultural production is facing unparalleled abiotic stress from global climate change (Ainsworth and Ort, 2010) with warming growing season temperatures the greatest threat (Vogel *et al*., 2019). Average terrestrial temperatures have increased ~1.4 °C since 1970 and are projected to increase an additional 1.2 °C by midcentury and 3.4 °C by 2100 if greenhouse gas concentrations continue to increase in the atmosphere at the current pace (Collins *et al*., 2013; Schwalm *et al*., 2020; Tebaldi *et al*., 2006). Increased warming over growing regions has already resulted in global yield losses of major grain crops, such as wheat, maize, rice and soybean (Lobell *et al*., 2011; Ray *et al*., 2019; Zhao *et al*., 2017), due to a combination of effects, including greater evaporative demand (Abtew and Melesse, 2013; Kimball and Bernacchi, 2006) and direct interference in both reproductive (Hedhly *et al*., 2009) and vegetative (Bita and Gerats, 2013; Dusenge *et al*., 2019; Moore *et al*., 2021; Posch *et al*., 2019; Slattery and Ort, 2019) physiological processes. Given that future warming scenarios predict greater heat stress in agricultural areas of both high‐ and low‐income countries, adaptation strategies are critical (Bailey‐Serres *et al*., 2019; Bita and Gerats, 2013; Deryng *et al*., 2014; Teixeira *et al*., 2013; Tilman *et al*., 2011).

Greater than 80% of the world’s most important crop plant species have C3 photosynthetic metabolism (Ray *et al*., 2019; Tilman *et al*., 2011). The most significant direct effect of temperature on photosynthesis in C3 plants is the increase in the ratio of ribulose‐1,5‐bisphosphate carboxylase‐oxygenase (Rubisco)‐catalysed oxygenation versus carboxylation of ribulose‐1,5‐bisphosphate (RuBP). Whereas the Rubisco carboxylation reaction results in two molecules of three‐carbon phosphoglycerate (PGA), Rubisco oxygenation produces one PGA and one molecule of two‐carbon P‐glycolate (2PG), which cannot re‐enter the photosynthetic carbon reduction cycle. Photorespiration recovers 75% of the photosynthetically reduced carbon in 2‐PG but at a cost of 3.5 ATP and 2 NADH equivalents per oxygenation reaction (Walker *et al*., 2016c). The remaining 25% of previously fixed carbon is released as CO_2_ in the mitochondria (Ogren, 1984). Elevated temperatures both reduce the intrinsic CO_2_ specificity of Rubisco and decrease the solubility of CO_2_ relative to O_2_ in the chloroplast, such that the oxygenation of RuBP by Rubisco is increasingly favoured as temperatures rise (Badger and Andrews, 1974).

In the United States, current rates of photorespiration are estimated to impose yield penalties of 20%–40% in wheat and soybean, and these photorespiratory rates and yield penalties are projected to increase with higher temperatures and under drought conditions (Walker *et al*., 2016c; Wingler *et al*., 1999). The ‘fertiliser effect’ of elevated [CO_2_] on C3 plant growth can largely be explained by reduced rates of photorespiration due to direct competition of the two substrates at the active site of Rubisco (Ainsworth and Long, 2005). As such, rising [CO_2_] will likely mitigate photorespiratory losses, but accompanying increases in growing temperature will continue to impose a significant yield penalty (Ruiz‐Vera *et al*., 2013). Models of photorespiratory losses in wheat and soybean under two future climate projections suggest that yield penalties of 8%–20% would persist at [CO_2_] of 600 ppm accompanied by a 3.7 °C increase in temperature (Walker *et al*., 2016c).

Lowering the cost of photorespiration in crops remains an important bioengineering target to improve crop yields (Bailey‐Serres *et al*., 2019; Eisenhut *et al*., 2019; South *et al*., 2019). Recently, we demonstrated large end‐of‐season dry biomass increases in tobacco plants expressing a synthetic alternative pathway (AP) to photorespiration that metabolises glycolate in the chloroplast (South *et al*., 2019). We hypothesised that the benefits of the introduced biochemical bypasses to the native photorespiration pathway will increase with increased photorespiratory pressure at elevated temperature. Here, we tested the physiological responses of tobacco plants expressing a synthetic AP when exposed to short‐term elevated temperature in controlled growth environments and long‐term elevated temperature when grown under warming conditions in the field. Overall, we found that the introduced AP increased the thermotolerance of net photosynthesis and mitigated end‐of‐season biomass loss in tobacco grown under elevated temperatures in agriculturally relevant conditions.

## Results

### APs to photorespiration are photoprotective at elevated temperature

**Figure 1:**
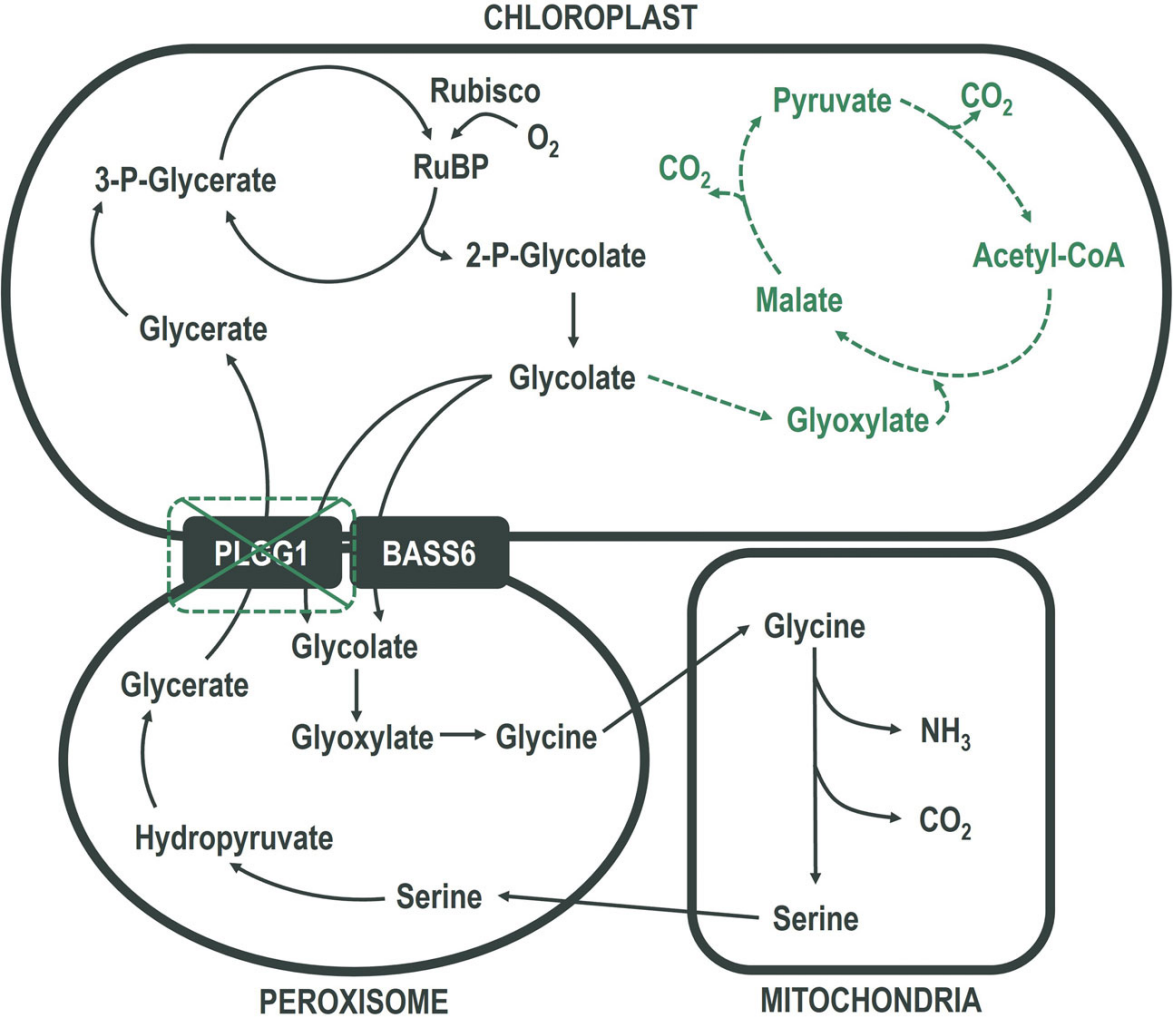
Pathway schematic of native and alternative photorespiration pathways. Alternative glycolate metabolism pathway (AP3) schematic. Chloroplast, peroxisome, and mitochondria based enzymatic reactions or transport steps of the native photorespiratory pathway (grey, solid lines) and AP3 (green, dashed lines) are shown. In AP3, the native glycolate‐glycerate transporter PLGG1 is downregulated via RNAi suppression to increase metabolic flux through the alternative metabolic pathway.

**Figure 2:**
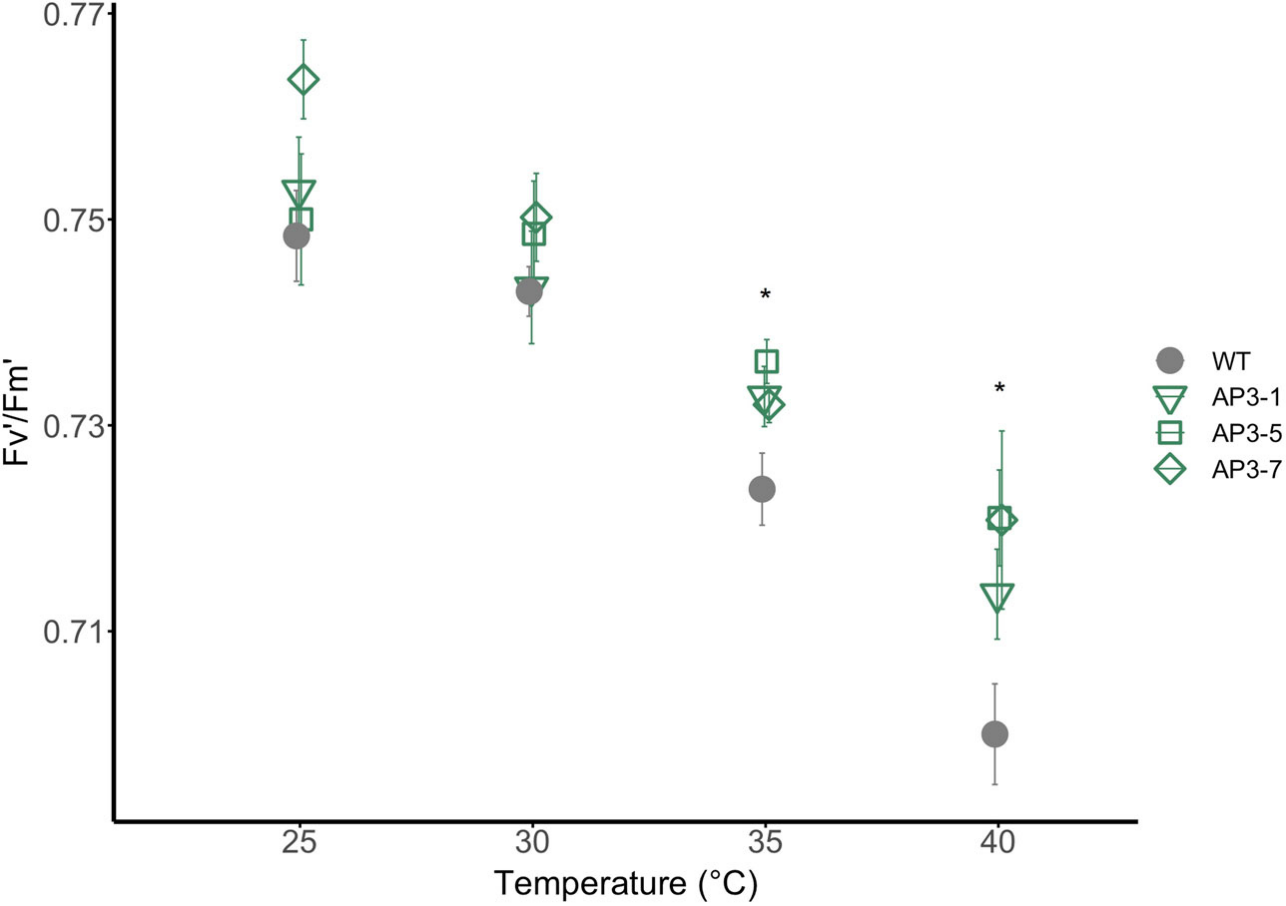
Photosynthetic operating efficiency ($F_{v}^{\prime }/F_{m}^{\prime }$) with temperature in chamber‐grown AP3 and WT lines. Values are from three independently transformed AP3 construct designs with RNAi targeting the glycolate‐glycerate transporter PLGG1 measured under temperature stress. Error bars indicate SEM. *Statistical difference in all AP3 lines compared to WT based on one‐way repeated measures ANOVA at *P* < 0.05; *n* = 6.

Alternative pathway function is photoprotective under high photorespiratory stress (i.e., low [CO_2_] and high light) (South *et al*., 2019). Since elevated temperature increases the frequency of Rubisco oxygenations, thereby increasing the rate of photorespiratory flux, we hypothesised that the AP function would also have a photoprotective effect at elevated temperature. To test this, we grew three AP tobacco lines (hereafter referred to as ‘AP3’ lines) independently transformed to overexpress genes for plant malate synthesis (MS) and *Chlamydomonas* glycolate dehydrogenase (CrGDH) in chloroplasts (Figure 1). The AP lines also expressed an RNA interference (RNAi) module targeting for reduced expression of the gene for a plastidic glycolate‐glycerate transporter (PLGG1) to lower export of glycolate from the chloroplast into the native pathway. We exposed the plants to four successive 24 h temperature regimes ranging from 25 to 40 °C in environmental growth chambers and measured photosystem II operating efficiency (i.e., $F_{v}^{\prime }/F_{m}^{\prime }$) at ambient temperature and at the end of each temperature treatment. AP3 lines exhibited higher $F_{v}^{\prime }/F_{m}^{\prime }$ values compared to the wild type (WT) at temperatures above 30 °C (Figure 2), indicating the AP enhances photoprotection by mitigating photodamage to photosystem II.

### AP3 enhanced net photosynthetic carbon assimilation at temperatures above the thermal optima

**Figure 3:**
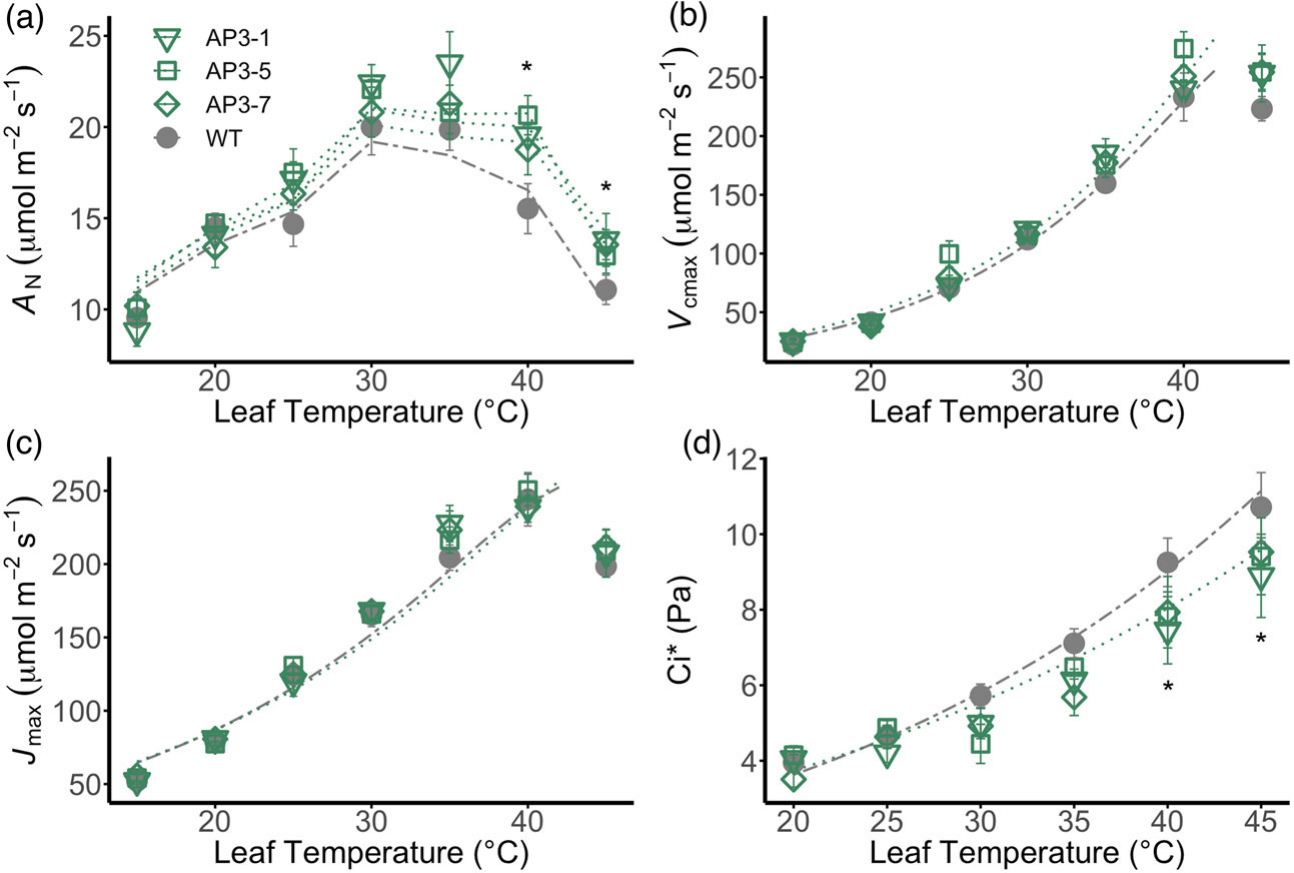
Temperature responses of photosynthetic parameters measured in greenhouse‐grown AP3 and WT lines. (a) Net CO_2_ assimilation at 400 ppm CO_2_. (b) Maximum rate of Rubisco carboxylation *V*_cmax_. (c) Maximum electron transport rate (*J*_max_). (d) Apparent CO_2_ compensation point ($C_{i}^{*}$) calculated using the common intercept method and slope regression (Walker *et al*., 2016a). Dotted (AP3) and dashed (WT) lines represent the modelled temperature response of each parameter based on either the calculated activation energy for each parameter (b–d) or by modelling the temperature response of photosynthesis according to the Farquhar‐von Caemmerer‐Berry model using parameters derived here (a). Data are the mean and standard error for *n* = 4–6 replicates. *Statistical differences at *P* < 0.05 between all AP3 lines and WT based on repeated measures ANOVA and Dunnett’s *post‐hoc* comparison.

Using the same AP3 lines as above, we examined responses of leaf photosynthetic rates and parameters of plants grown in the greenhouse and exposed to temperatures ranging from 15 to 45 °C. All lines had similar rates of photosynthesis at temperatures leading up to peak photosynthetic rates (i.e., the thermal optimum). However, at temperatures above the thermal optimum, where Rubisco more strongly favours oxygenation over carboxylation, the AP3 lines maintained higher photosynthetic rates (Figure 3a). At 40 °C, AP3 CO_2_ fixation rates were 5%–18% higher than the WT among the three independent transgenic lines (Figure 3a). These differences were not related to differences in apparent Rubisco carboxylation capacity (i.e., *V*_cmax_; Figure 3b) or rates of electron transport (i.e., *J*_max_; Figure 3c; Figure S2), which did not vary between transgenic and WT plants at any measurement temperature. At higher temperatures, the intercellular [CO_2_] at the CO_2_ compensation point ($C_{i}^{*}$) was lower in transgenic plants than in WT plants (Figure 3d; Table S2).

### Synthetic AP3 pathway functioned at both ambient and elevated temperature in the field

**Figure 4:**
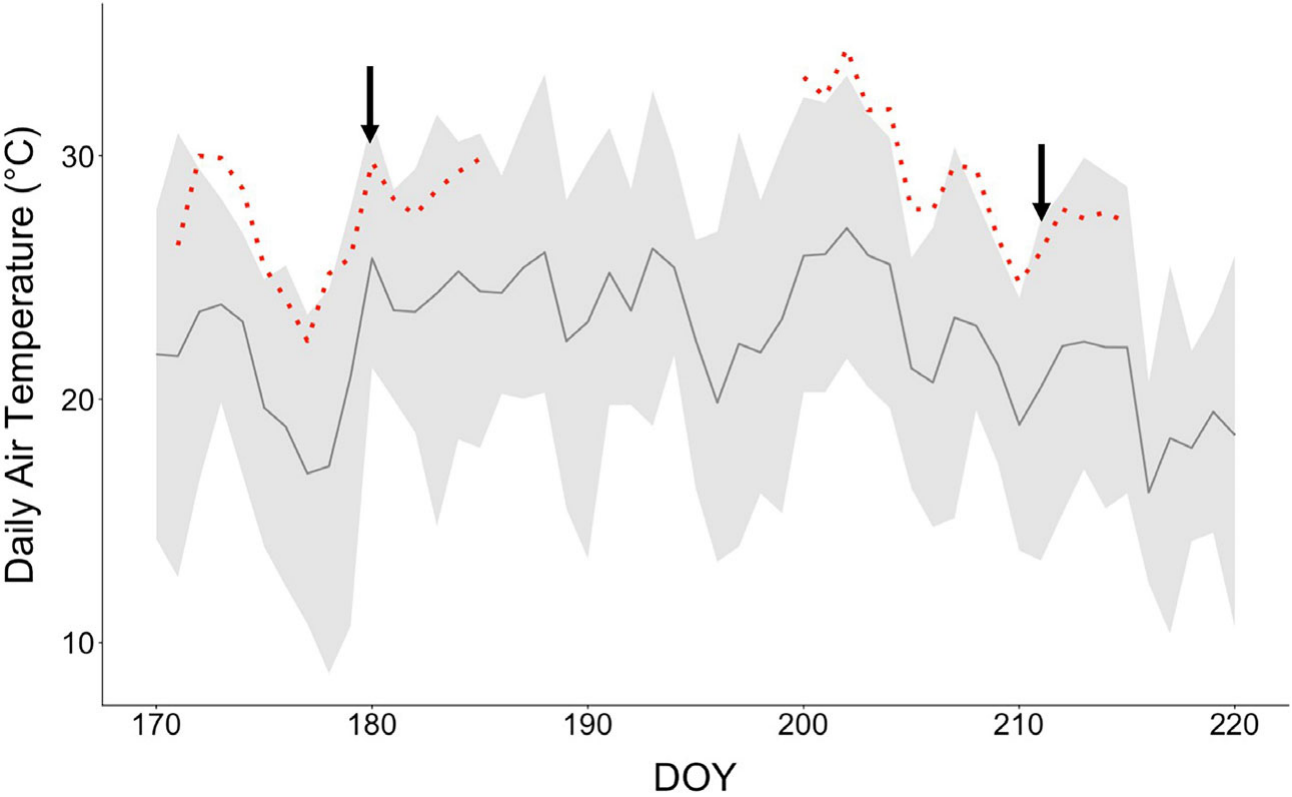
Daily air temperature of ambient and heated plots throughout the duration of two field‐warming experiments. Solid grey line represents the mean and shaded grey regions represent the daily max‐min air temperature with DOY. The average daily canopy temperature of heated plots for each experiment is plotted in the dotted red. Black arrows indicate the DOY of *in situ* diurnal measurements of photosynthesis in each experiment.

In the 2017 growing season, we tested the three independently transformed AP3 lines and an azygous WT control under field heating conditions in two successive experiments. Daily mean air temperature was 22.2 ± 2.8 °C for the field experiment planted on June 12, 2017 (day of year, DOY 163) and 23.6 ± 3.0 °C for the field experiment planted on July 12, 2017 (DOY 193). Canopy heat treatments began DOY 170 for experiment 1 and 200 for experiment 2 and targeted a 5 °C increase in canopy temperature as compared to ambient. Over the warming period, actual canopy temperature increased by 4.8 and 4.6 °C compared to ambient canopy temperature, resulting in mean heated canopy temperatures of 27.4 ± 2.4 °C (ambient 22.6 ± 2.4 °C) in the first experiment, and 29.1 ± 2.8 °C (ambient 24.2 ± 2.4 °C) in the second (Figure 4).

**Figure 5:**
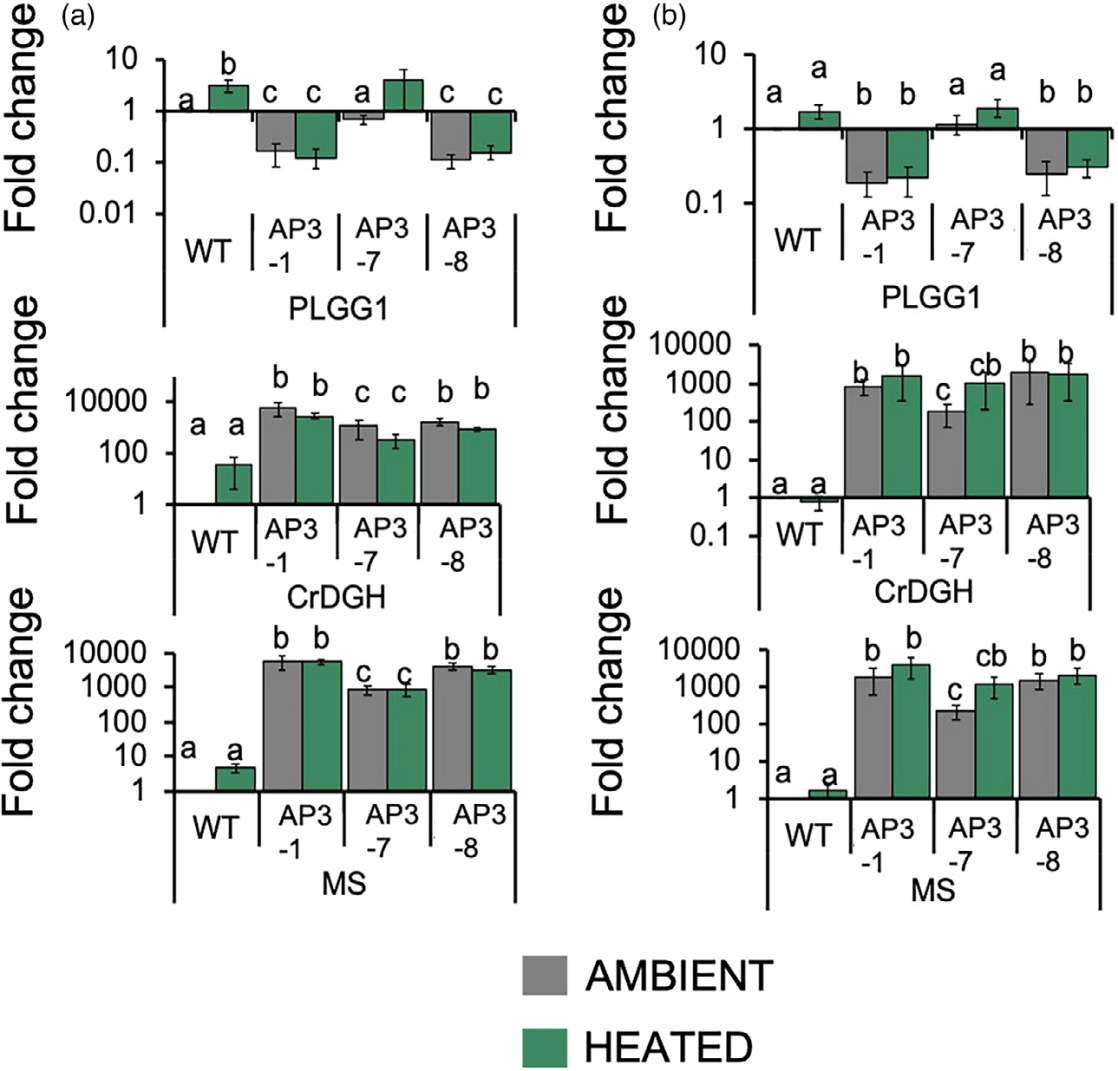
Gene expression in field‐grown AP3 and WT lines under ambient and heated conditions. qRT‐PCR analysis of the two transgenes in AP3 and the target gene *PLGG1* of the RNAi construct under ambient (grey) and heated (green) treatments for field‐grown plants in the first (a) and second (b) experiment of the season. Results for four (experiment 1) and three (experiment 2) plots are shown. Error bars indicate standard error. Letters indicate statistical difference at *P* < 0.1 based on one‐way ANOVA followed by Tukey’s HSD *Post‐hoc* test.

Expression analysis from field‐grown plants confirmed *MS* and *CrGDH* were highly expressed at in all AP3 lines, and transgene expression did not vary with temperature in either experiment (Figure 5). However, one independent AP line (AP3‐7) showed lower transgene expression levels than the other two. RNAi suppressed *PLGG1* expression by ~75% in both ambient and elevated temperature conditions in two transgenic lines (AP3‐1 and AP3‐8) but not in AP3‐7 (Figure 5). In the first experiment, both AP3‐7 and WT plants grown at elevated temperatures increased *PLGG1* gene expression by ~5%, indicating that *PLGG1* expression is slightly upregulated in response to growth at elevated temperatures (Figure 5).

### AP3 plants showed increased quantum efficiency and daily carbon gain under agriculturally relevant conditions

**Figure 6:**
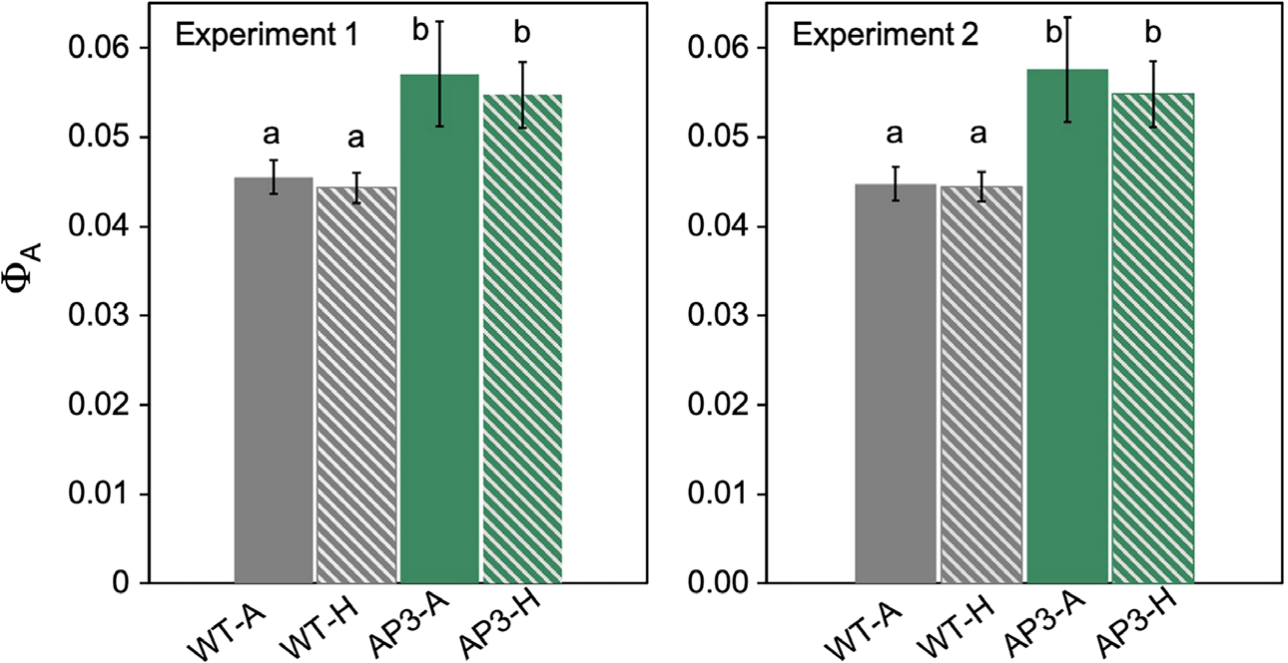
Apparent quantum efficiency of photosynthesis (Φ_A_) of field‐grown AP3 and WT lines in ambient and heated conditions. Φ_A_ was determined by linear regression of assimilation based on light‐response curves below 200 μmol/m^2^/s for WT (grey) and AP3 (green) plants grown under ambient (solid) and heat (dashed) treatment. Data were the combined result of AP3 transformants, and results for four (experiment 1) and three (experiment 2) plots are shown. Error bars represent standard error, and letters represent statistical difference at *P* < 0.1 based on Dunnett’s *post‐hoc* comparisons following mixed effects model analysis.

AP3 plants have improved maximum quantum efficiency of net CO_2_ assimilation (Φa) compared to WT plants (30), and we hypothesised that this benefit would increase under elevated temperature conditions. Here, field‐grown AP3 lines showed 25% greater Φa than WT plants under non‐heated conditions (25% in experiment 1, 28% in experiment 2) and 23% greater Φa than WT plants under heated conditions, indicating that AP3 plants maintained a similar energetic advantage of CO_2_ fixation at elevated temperatures (Figure 6; Figure S3).

**Figure 7:**
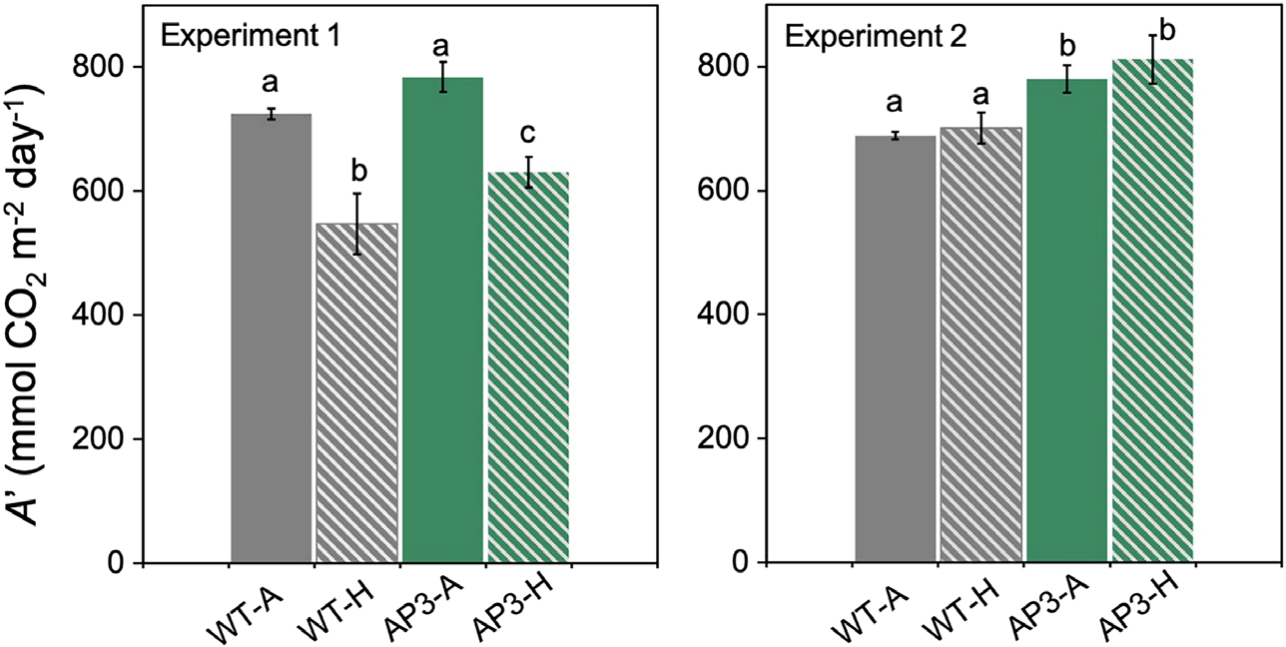
Accumulated assimilation of CO_2_ (*A*′) in field‐grown AP3 and WT lines in ambient and heated conditions. *A*′ was determined based on diurnal analysis of photosynthesis for WT (grey) and AP3 (green) plants grown under ambient (solid) and heat (dashed) treatment. Data were the combined result of AP3 transformants and results for four (experiment 1) and three (experiment 2) plots are shown. Error bars represent standard error, and letters represent statistical difference at *P* < 0.1 based on Dunnett’s *post‐hoc* comparisons following mixed effects model analysis.

Above‐optimal growth temperatures result in lower total daily net carbon gain (Dusenge *et al*., 2019; Sage and Kubien, 2007; Slattery and Ort, 2019; Walker *et al*., 2016c; Wu *et al*., 2018); thus, we hypothesised that this loss would be mitigated in plants expressing AP3. Daily net carbon gain (*A*′; estimated from measurements of photosynthesis over a diurnal time course as in Bernacchi *et al*., 2006) in AP3 lines increased by 17% compared to WT under heated conditions when measured in experiment 1 and by 16% in experiment 2 (Figure 7). In the first experiment, this was driven by a growth temperature effect that lowered *A*′ by 25% in WT plants, but only 20% in AP3 plants. In the second experiment, mean ambient temperature was 1.6 °C higher than the first, but this temperature effect was not detected, and *A*′ did not differ between heated and ambient treatments in WT or AP3 plants. No change was detected in electron use in photosynthesis (*J*′) compared to WT under either growth condition, which is consistent with the lack of differences in whole electron chain transport measured in greenhouse plants at any temperature (Figures S2 and S4).

### AP3 conferred thermal protection for growth under agriculturally relevant conditions

**Figure 8:**
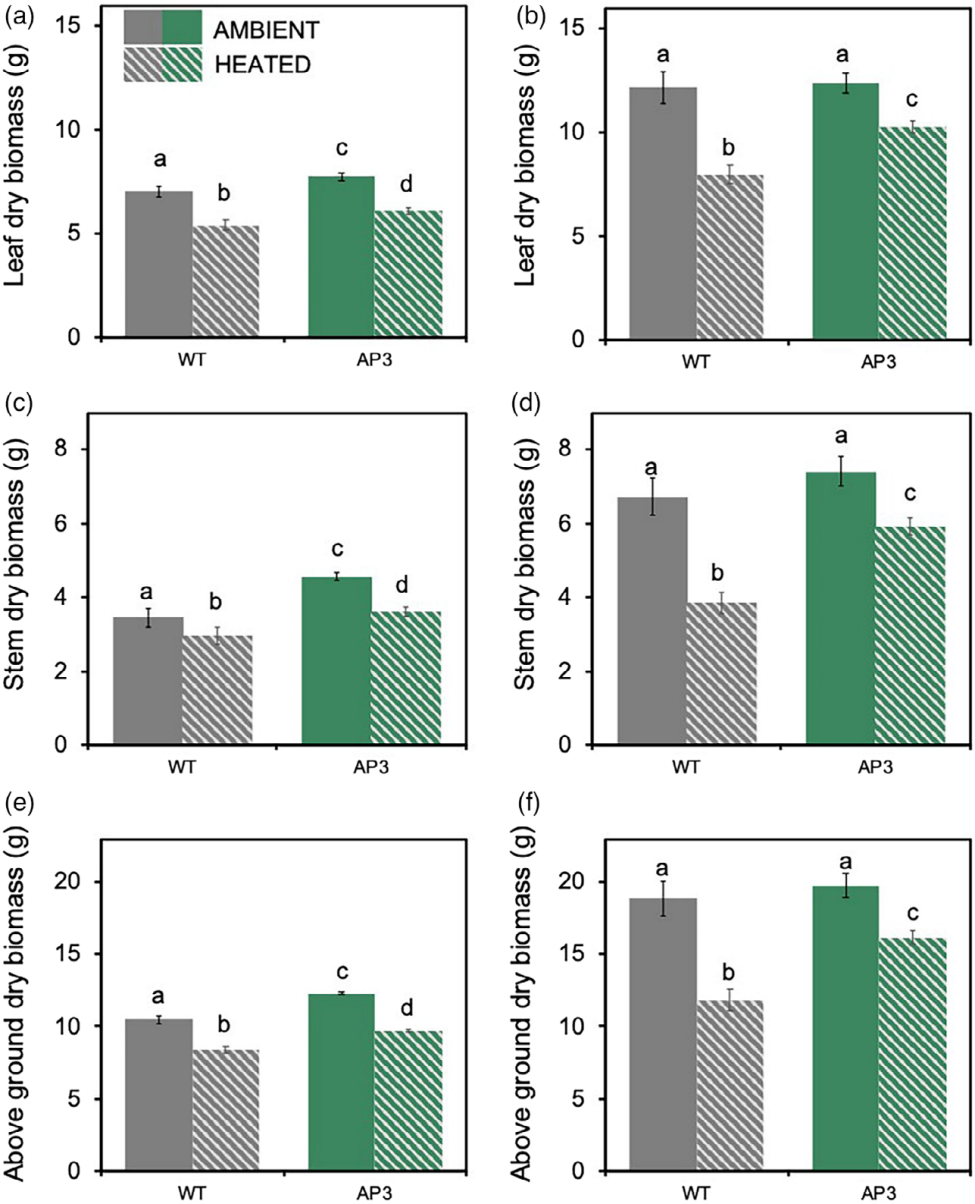
Dry weight biomass of field‐grown AP3 and WT lines under ambient and heated conditions. Leaf (a, b), stem (c, d), and total (e, f) biomass of AP3 (green) and WT (grey) plants grown under ambient (solid) and heat (dashed) treatment from plantings 1 (a, c, e) and 2 (b, d, f). Data were the combined result of AP3 transformants and results for four (experiment 1) and three (experiment 2) plots are shown. Error bars represent standard error, and letters represent statistical difference at *P* < 0.1 based on Dunnett’s *post‐hoc* comparisons following mixed effects model analysis.

**Figure 9:**
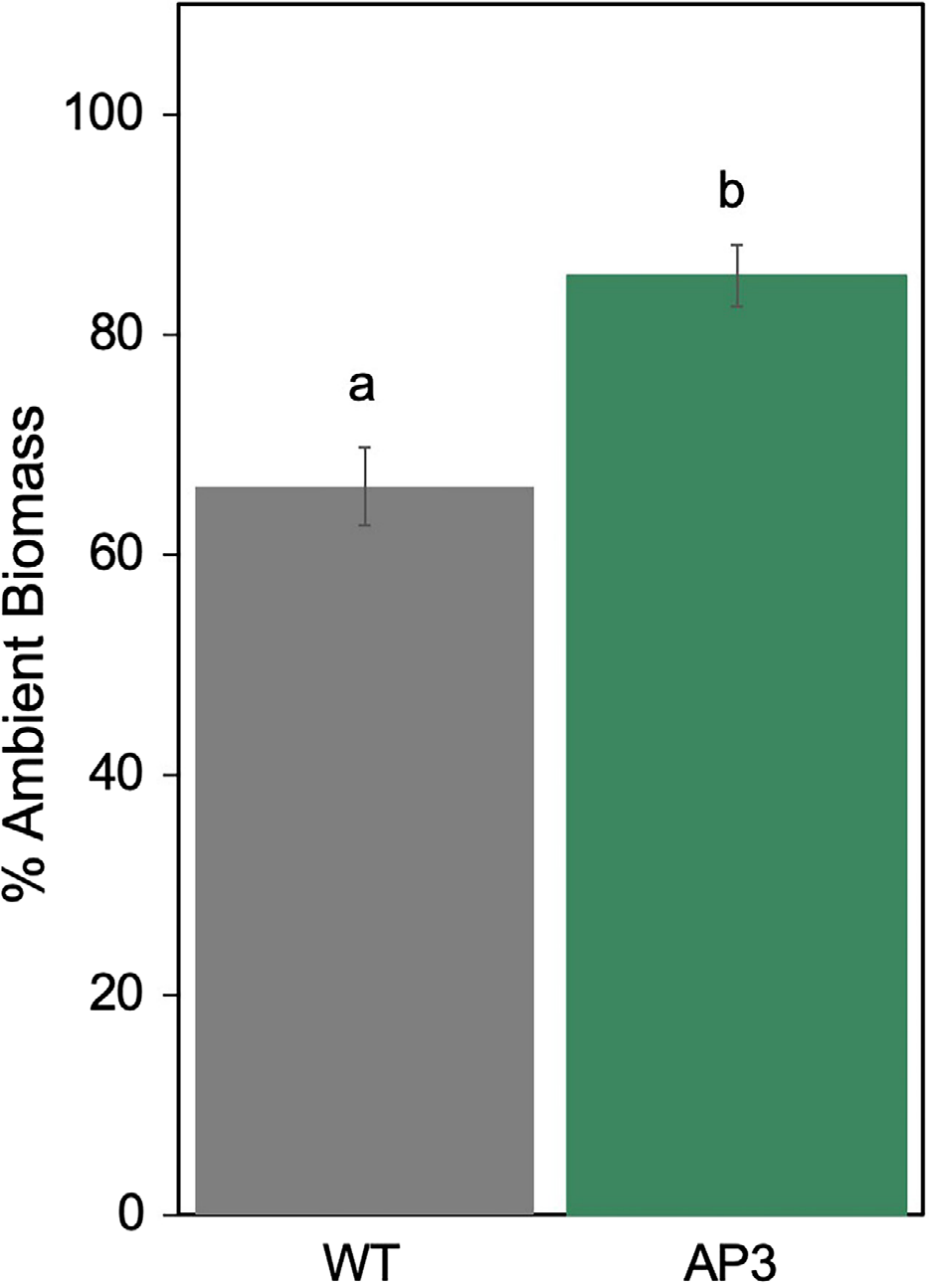
Dry weight biomass retained under heating conditions relative to ambient conditions. Mean responses of WT (grey) and AP3 (green) plants were the combined result of seven experimental plots over two planting experiments. Error bars represent standard error, and letters represent statistical difference at *P* < 0.1 based on Dunnett’s *post‐hoc* comparisons following mixed effects model analysis.

In the first experiment, end‐of‐season total dry‐weight biomass of transgenic plants was 16% greater than WT under ambient conditions (9% leaf, 32% stem), and 17% greater than WT under heated conditions (12% leaf, 22% stem; Figure 8). Growth at elevated temperature reduced total biomass by 28% in WT plants but only by 18% in AP3 plants relative to the respective ambient controls (Figure 8; Figure S5). In the second experiment, total above ground biomass was ~2/3 greater than in the first, and there was no significant effect of AP3 on biomass production in ambient conditions. Canopy warming during this period reduced WT biomass by 38% and transgenic biomass by 23%, such that total dry‐weight biomass of AP3 was 37% greater than WT (28% leaf and 54% stem) under heated conditions. Over both experiments AP3 plants retained 85% of total ambient biomass under warming conditions, while WT plants retained 66% (Figure 9).

## Discussion

The objective of this study was to determine the potential for synthetic photorespiratory pathways installed entirely within chloroplasts to confer thermotolerance in C3 plants. This complements and extends our previous work, which demonstrated that modified photorespiratory metabolism improved growth and performance of field‐grown tobacco (South *et al*., 2019). In this study we found that the AP3 pathway enhanced photosynthetic performance in both short‐term warming in controlled environments as well as growth under long‐term canopy warming under field conditions. The increased thermostability of AP3 tobacco plant photosynthesis was associated with greater end‐of‐season biomass accumulation relative to WT at elevated temperature, suggesting a greater benefit of AP3 at elevated temperatures. Ultimately this enhanced benefit under heated conditions resulted in transgenic plants retaining 19% more biomass relative to WT controls, consistent with expectations from photosynthetic gas exchange theory that the benefit of more energetically efficient glycolate recycling pathways would be greater at higher temperatures where the production of glycolate would be greater.

Overexpression of native photorespiratory pathway components can confer improved thermal tolerance (Cui *et al*., 2016; Timm *et al*., 2019), but redirecting photorespiratory glycolate flux through APs has not been investigated in this regard. Photorespiratory flux must increase with rising temperature to account for increases in the Rubisco oxygenation rate. Thus increased release of previously assimilated CO_2_, a corresponding increase in the CO_2_ compensation point and decreased quantum efficiency of photosynthesis resulting in lower net carbon gain for a given amount of photons absorbed are associated with higher temperature (Ehleringer and Björkman, 1977; von Caemmerer, 2000). Plants expressing AP3, which has previously shown enhanced performance under high photorespiratory stress (South *et al*., 2019), also demonstrated enhanced thermal protection (Figure 2) and higher rates of leaf photosynthesis above 35 °C (Figures 2 and 3a). Interestingly, we saw no increase in maximum carboxylation or electron transport rates in AP plants, despite the observation that decreased net assimilation in the Arabidopsis plgg1 mutant is caused by decreased Rubisco activity and electron transport (Walker *et al*., 2016b). Instead, the maintenance of higher photosynthesis in AP plants under short term temperature stress is associated with lower apparent intercellular CO_2_ partial pressures at the CO_2_ compensation point ($C_{i}^{*}$) (Figure 3d), supporting the hypothesis that the benefit conferred by the AP3 pathway is enhanced photorespiratory CO_2_ refixation in the chloroplast (Peterhansel *et al*., 2012; South *et al*., 2019; Tholen *et al*., 2012).

The advantage that AP3 chloroplastic CO_2_ release confers to net assimilation will depend upon existing level of refixation, which varies among C3 species (Busch *et al*., 2013). During photorespiration, one half mole of CO_2_ is released in the mitochondria for each oxygenation reaction catalysed by Rubisco in the chloroplast, but the stoichiometry of AP examined in this work suggest that instead, two moles of CO_2_ should be released per Rubisco oxygenation: first when glyoxylate is decarboxylated to pyruvate, and again upon oxidation of pyruvate to CO_2_ (Maier *et al*., 2012; South *et al*., 2019). Metabolic modelling of this AP strategy predicts that full decarboxylation of glycolate perform worse than the native photorespiration pathway (Xin *et al*., 2015). However, no change in the CO_2_ compensation point was found in Arabidopsis plants expressing a glycolate oxidising pathway, and reductions in the apparent compensation point have been demonstrated in both tobacco and rice plants employing this decarboxylating strategy (Figure 3d; Maier *et al*., 2012; Shen *et al*., 2019; South *et al*., 2019). This indicates increased CO_2_ availability in the chloroplast. Differences between tobacco and Arabidopsis could arise from species specific differences in mesophyll conductance (Tholen *et al*., 2012), or differences in the arrangement of chloroplasts inside the cell, which is known to impact photorespiratory carbon refixation ability (Busch *et al*., 2013). Increased chloroplast coverage of mesophyll cells enable photorespired CO_2_ to pass into chloroplasts, thereby raising the chloroplastic CO_2_ concentration. Notably, tobacco has a low trapping potential compared to other species, where about 60%–80% of the mesophyll cell wall area is covered by chloroplasts (Busch *et al*., 2013; Clarke *et al*., 2021; Evans *et al*., 1994), compared to 90% coverage in Arabidopsis (Xiong *et al*., 2017), and almost complete coverage in rice (Sage and Sage, 2009). Furthermore, this relationship can vary with plant development or growth condition (Clarke *et al*., 2021; Tosens *et al*., 2012), highlighting important considerations when moving AP engineering strategies out of model plants and into crop species.

Functioning photorespiratory metabolism is crucial for C3 to survive in an oxygenic atmosphere, and under fluctuating light conditions, strong activation of the photorespiratory pathway regulates photosynthetic electron flow, accelerating RuBP regeneration to maintain rates of CO_2_ fixation (Huang *et al*., 2015). However, in both controlled environment and field studies AP3 tobacco plant metabolism could compensate for reduced flux through the peroxisomal and mitochondrial parts of native photorespiration, displaying both higher quantum efficiency and higher daily carbon gain than the non‐transgenic controls (South *et al*., 2019). Here, we extended our previous work to investigate if the transgenic glycolate metabolic pathway might be thermoprotective, as rates of photorespiration increase with elevated growth temperature. Although increases in daily carbon gain in heated AP3 plants relative to WT controls were similar between experiments, the impact of temperature was inconsistent, depressing values of *A*′ in experiment 1, but not 2 (Figure 7). Between the first day of canopy warming (DOY 170 and 200) and *in situ* measurements of daily carbon assimilation (DOY 180 and 213), the average heated canopy temperature between transplant and *in situ* diurnal measurement was 26.4 °C in experiment 1, and 29.1 °C in experiment 2 (Figure 4). It is likely that canopy warming induced a greater depression in *A*′ in experiment 1 (DOY 180; heated *T*_can_ = 30.8 °C) than experiment 2 (DOY 213; heated *T*_can_ = 27.8) because the plants were acclimated to cooler growth temperatures. In this situation, enhanced photorespiratory refixation could compensate for biochemical or diffusional limitations of net photosynthesis which increasingly limit C3 crops as temperatures increase.

At elevated temperature, yield losses from increased photorespiratory costs are common to all C3 crops, though the impact varies by growing region (Lobell *et al*., 2011; Ray *et al*., 2019; Walker *et al*., 2016c). For example, increasing the mean temperature of field‐grown spring wheat by 1.7 °C results in an increase in net photosynthesis, while a 3.5 °C increase above ambient seasonal growth temperatures reduces net photosynthesis and yield in soybean during a warm, but not cool, growing season (Ruiz‐Vera *et al*., 2013; Wall *et al*., 2011). Similarly, climate change impacts, which are largely driven by increased temperature, are already exerting a considerable drag on C3 crop yields globally, with some exceptions for yield improvements in high latitude countries (Lobell *et al*., 2011; Ray *et al*., 2019; Zhao *et al*., 2017). Climate change has reduced global yields of wheat, rice, barley and cassava, with regional yield losses evident in soybean and rapeseed, and future temperature increases are predicted to lower global yields of wheat, rice and soybean (Ray *et al*., 2019; Zhao *et al*., 2017). Mitigation strategies are essential to maintain agricultural yields, and recent advances in plant synthetic biology and transformation potentially offer tools to help realise this (Bailey‐Serres *et al*., 2019; Chen *et al*., 2020; Zaidi *et al*., 2019). Here, we have demonstrated that engineering glycolate metabolisms is a potential strategy to boost C3 crop yield resilience to the increasing temperature stress conditions that climate change portends. Going forward, advances in synthetic biology will require development of modules such as this alternative glycolate metabolism to protect against future climate challenges, making stress testing genetic parts used in designs essential to meeting sustainability goals.

## Methods

### Plant material

This study used T2 transgenic lines of *Nicotiana tabacum* cv. ‘Petite Havana’ transformed with *Chlamydomonas reinhardtii* glycolate dehydrogenase, *Cucurbita maxima* malate synthase, and an RNAi module targeting the plastidic glycolate‐glycerate transporter *PLGG1* as previously described (South *et al*., 2019). WT plants used in this study were azygous plants, which had been through the transformation protocol, but did not contain the transgenic construct.

### Chlorophyll fluorescence measurements

Tobacco T_2_ seeds were germinated under ambient air conditions on soil in a controlled environment chamber (Environmental Growth Chambers, Chagrin Falls, OH) with 14 h day (25 °C)/10 h night (20 °C) and light intensity of 500 μmol/m^2^/s. Ten days after germination, growth temperature was subsequently increased every 24 h for five days by stepwise increments of 5 °C with the last treatment being 45 °C. Following the end of each thermal exposure, $F_{v}^{\prime }/F_{m}^{\prime }$ was determined using the CF Imager Technologica (http://www.technologica.co.uk/) as previously described (South *et al*., 2019). Briefly, $F_{v}^{\prime }/F_{m}^{\prime }$ images were taken after 5 min dark adaptation at room temperature (~24 °C) and ambient CO_2_. Maximum flash intensity was 6800 µmol/m^2^/s for 800 ms. Following each image, plants were returned to the controlled environment chamber, temperature was increased by 5 °C, and plants were re‐measured the following day.

### Gas exchange measurements (greenhouse)

Temperature responses of net CO_2_ assimilation were measured on the fifth leaf from the base of greenhouse‐grown 7‐week‐old *N*. *tabacum* plants using a Li‐Cor 6800 infrared gas analyser (Li‐Cor Biosciences, Lincoln, NE). Leaf temperature was controlled at the set temperature, and measurements were conducted in a growth chamber (Conviron) for temperature control. Photosynthesis was measured at a PPFD of 1800 μmol/m^2^/s to ensure saturation of photosynthesis at all measurement temperatures. For photosynthetic [CO_2_]‐response curves, leaves were acclimated at a [CO_2_] of 400 ppm to achieve a steady‐state rate of assimilation. The [CO_2_] of the response curve was set at 400, 200, 150, 100, 75, 50, 30, 400, 600, 800, 1000, 1500, 2000 ppm, and measurements were taken when assimilation reached a steady state rate at each [CO_2_] setting. To determine the maximum rate of carboxylation (*V*_cmax_) and maximum electron transport rate (*J*_1800_), a model for leaf photosynthesis with temperature corrections was used assuming infinite mesophyll conductance (Bernacchi *et al*., 2001). Following each *A*/*C*_i_ curve measurement, leaves were allowed to reach steady state, and the measurements of *R*_d_ and $C_{i}^{*}$ were determined using the common intercept method, which takes advantage of changes in photorespiration at different PPFD (Walker *et al*., 2016a).

### Field site description and experimental conditions

Effects of elevated temperature conditions on field grown AP3 plants were evaluated twice in a complete block design (Experiment 1, *n* = 4; Experiment 2, *n* = 3) at an experimental field site in the SoyFACE field facility near Urbana‐Champaign, IL (40°2′30.49″N, 88°13′58.80″W, 230 m above sea level). The field experiment consisted of four blocks, each containing one ambient and one heated plot consisting of 12 × 12 plants spaced 23 cm apart (Figure S1). In the second experiment, replication decreased to three blocks due to a lethal flooding event in one block. Each plot was further divided into quadrants of four 6 × 6 subplots of WT and three independent AP3 lines, with genotypes arranged randomly among subplots (Figure S1). Single insert homozygous T2 seeds of AP3 lines and azygous WT plants were sown on growing medium (LC1 Sunshine mix, Sun Gro Horticulture, Agawam, MA) in the greenhouse on DOY 131 and 167. After 14 days, plants were transferred to floating trays for hydroponic growth for two weeks as described (South *et al*., 2019). Seedlings were transplanted to the experimental field on DOYs 163 and 193 after the field was prepared as previously described (Kromdijk *et al*., 2016). Watering was provided as needed through parallel drip irrigation lines. The heated subplots were each equipped with an infrared heater array maintained at 1.0 m above the top of the canopy on a telescopic mast system described previously (Ruiz‐Vera *et al*., 2013). Each total subplot area was 7.5 m^2^, and eight internal plants/genotype (Figure S1) were used for physiology and biomass measurements. Using a proportional‐integral‐derivative feedback control system, the canopy was warmed continuously day and night to a target elevation of +5 °C above the canopy temperature in the unheated plot beginning DOYs 170 and 200. Canopy temperature under each heating array was measured via an infra‐red radiometer (IRR; SI‐111; Apogee Instruments, Logan, UT) connected to data‐loggers (CR1000 Micrologger; Campbell Scientific, Logan, UT).

### Field measurements

Apparent quantum efficiency of photosynthesis (Φa) and the light‐saturated rate of photosynthetic CO_2_ assimilation were measured on the youngest fully expanded leaf on DOYs 173–176 and 212–214. Gas exchange measurements were performed using Li‐Cor 6400XT instruments with a 2‐cm^2^ fluorescence measuring cuvette for which chamber leaks were corrected as outlined in the manual (LI‐COR Biosciences). Measurements of CO_2_ assimilation were conducted on dark adapted leaves at incident light intensities of 0, 15, 30, 40, 50, 70, 90, 115, 150, 300, 600, 1200, and 2000 μmol/m^2^/s PPFD, and absorbed light was calculated using an integrating sphere (OceanOptics, Largo, FL). Leaf temperature was set to 30° for ambient and 35 °C for heat‐treated plants. Assimilation was recorded after a minimum of 180 s at each light level. The slope of the initial response of CO_2_ assimilation at low light levels (i.e., <200) was examined for linearity and used to calculate Φa. Diurnal measurements of photosynthesis were performed starting pre‐dawn on DOY 180 and 218 and measured every 2 h on two plants per line per treatment per block. Light levels and chamber temperature were set to ambient values based on incoming light levels using the PPFD sensor on the Li‐Cor 6400XT and the canopy temperature reading from the IRR associated with each plot. Reference [CO_2_] was maintained at 400 ppm. Diurnal measurements were continued until after dusk. At 54 (experiment 1) and 52 (experiment 2) days post‐germination, eight plants per plot were harvested from all four (experiment 1) and three (experiment 2) replicate blocks. Aboveground biomass was separated into leaf and stem fractions and dried at 65 °C to constant weight for a minimum of 2 weeks prior to dry biomass measurements. Proportion of biomass retained under heating conditions for WT and transgenic plants was calculated per plot by dividing plant dry weight biomass by the mean value of the corresponding ambient plot.

### Gene expression analysis

Approximately 100 mg of leaf material was harvested from three plants per line per treatment per block, flash frozen in liquid nitrogen and stored at −80 °C. RNA was extracted using the Nucleospin RNA kit (Macherey‐Nagal GmbH& Co.KG, Düren, Germany). cDNA was generated using the Qiagen Quantinova reverse transcription kit. Gene expression analysis was performed on a Bio‐Rad CFX connect Real‐Time PCR system. Relative changes in gene expression were determined using the *L25* gene as a reference using three technical replicates per biological sample. Amplification was performed using the Bio‐Rad SSO advanced SYBR green master mix, and relative levels were determined using the ΔΔCT method. Primer sequences used are described in Table S1.

### Statistical analysis

$F_{v}^{\prime }/F_{m}^{\prime }$ measurements were analysed as a repeated measures ANOVA with five replicate plants per line over each measurement temperature. Greenhouse photosynthetic measurement data were analysed by a repeated measures two‐way ANOVA testing effects of pathway and measurement temperature, using six biological replicates per measurement, followed by a Dunnett’s *post‐hoc* test for means comparison, and data were considered significant at *P* < 0.05. The activation energy for temperature responses of $C_{i}^{*}$ were fit using non‐linear least squares regression using the R package minpack.lm, compared using a one‐way ANOVA, and used to model the temperature response of photosynthesis according to the Farquhar‐von Caemmerer‐Berry model (Farquhar *et al*., 1980) when activation energy differed from WT plants.

Both field experiments were analysed separately as a split‐plot design using a mixed‐model ANOVA design with temperature (ambient and heated) and genotype (WT and AP3) and their interactions as fixed effects and block as a random effect. The impact of canopy heating on plot biomass losses was analysed over both rounds using a linear mixed model that included temperature (ambient and heated) and transgenic pathway (WT and AP3), independent line and their interactions as fixed effects and experiment as a random effect. Following mixed‐model analysis using the CRAN package ‘lme4’, least square means were compared using a *post‐hoc* Dunnett’s comparisons when the model was significant using the CRAN package ‘eemeans’. Because of the small sample size in the field experiment (*n* = 4 experiment 1; *n* = 3 experiment 2), α was set *a priori* to 0.1 in field analysis to minimise the potential for type II errors as is standard for this field site (http://www.soyface.uiuc.edu). Statistical analysis was performed in R (version 3.4.2 https://www.R‐project.org/).

## Conflict of interest

The authors declare no conflicts of interest.

## Author contributions

All authors conceived of and designed the original research plans. CJB developed and validated field infrastructure and curated heating data. APC and PFS sampled field plants and measured physiological responses. APC performed quality control and statistical analysis. APC and DRO wrote the article with contribution from all authors.

## Supporting information


**Figure S1** Representative plot used in the 2017 field experiment.
**Figure S2** Temperature responses of photosynthetic electron transport rate measured in greenhouse‐grown AP3 and WT lines.
**Figure S3** Photosynthetic parameters estimated from light response curves.
**Figure S4** Accumulated assimilation of CO_2_ (*A*′) in field‐grown WT and three independent transformations of AP3 in ambient and heated conditions.
**Figure S5** Dry weight biomass of field‐grown WT and 3 independent transformations of AP3 under ambient and heated conditions.
**Figure S6** Dry weight biomass retained under heating conditions relative to ambient conditions.
**Table S1** A list of primers used in this work
**Table S2** The scaling constant (*c*) and heat of activation (Δ*H*_a_) of $C_{i}^{*}$ measured in WT and AP3 plants
