# Supplementary material for: Alternative pathway to photorespiration protects growth and productivity at elevated temperatures in a model crop
Source: Plant Biotechnol J. 2021 Nov 24;20(4):711–21. doi: 10.1111/pbi.13750 (PMC8989507; doi:10.1111/pbi.13750)
Supplement: Supplementary file 1 — Figure S1 Representative plot used in the 2017 field experiment. Figure S2 Temperature responses of photosynthetic electron transport rate measured in greenhouse‐grown AP3 and WT lines. Figure S3 Photosynthetic parameters estimated from light response curves. Figure S4 Accumulated assimilation of CO2 (A′) in field‐grown WT and three independent transformations of AP3 in ambient and heated conditions. Figure S5 Dry weight biomass of field‐grown WT and 3 independent transformations of AP3 under ambient and heated conditions. Figure S6 Dry weight biomass retained under heating conditions relative to ambient conditions. Table S1 A list of primers used in this work Table S2 The scaling constant (c) and heat of activation (ΔH a) of Ci∗ measured in WT and AP3 plants [file PBI-20-711-s001.pdf]

Figure S1

A B

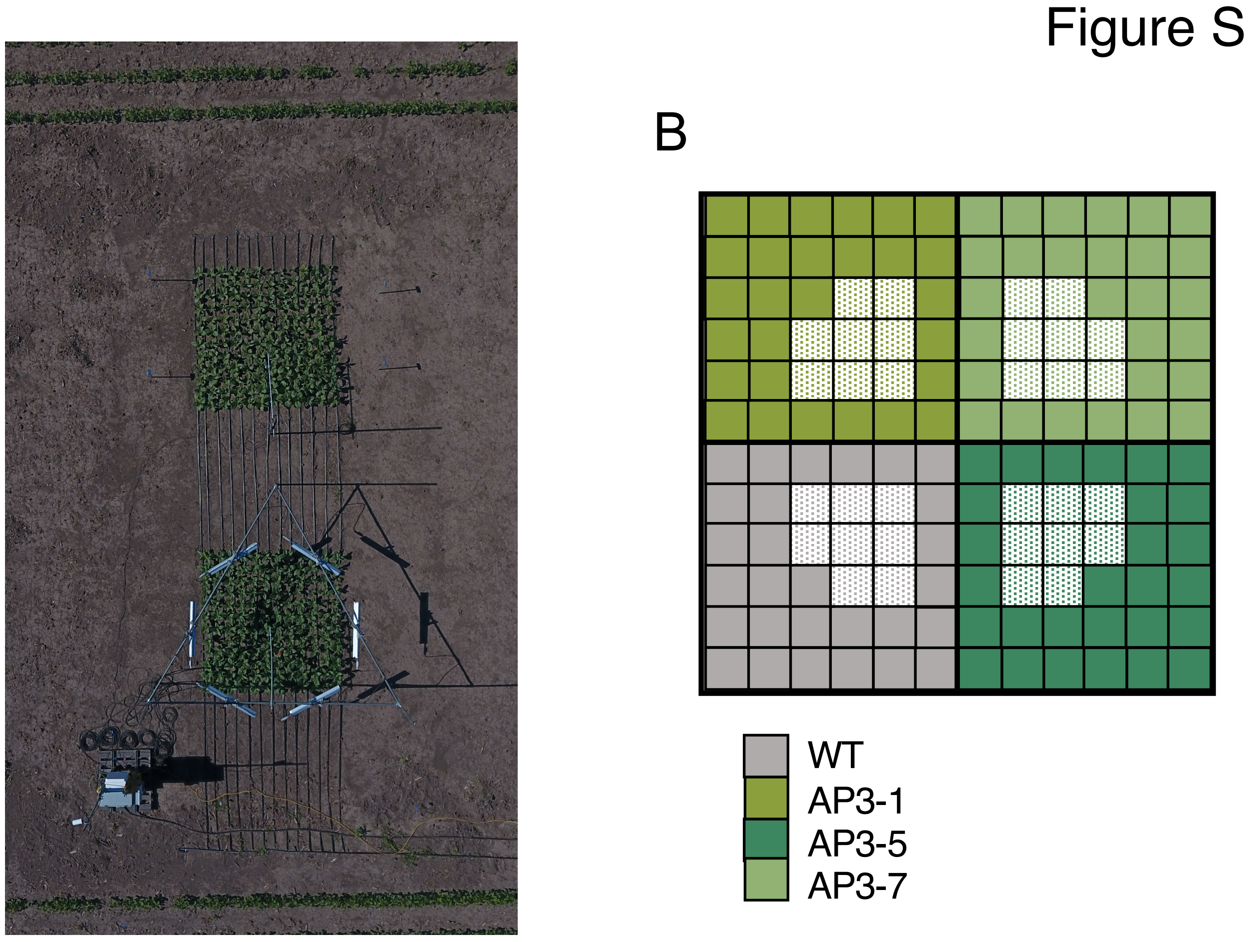

Supplemental Figure 1: Representative plot used in the 2017 field experiment. A) Representative block consisting of one heated (lower, shown with heater arrangement) and one ambient (upper) sub-plot. B) Each subplot contained 144 plants further subdivided into 4 groups of 36 plants: azygous WT (gray), and three independent AP3 lines (green). Genotypes were arranged randomly among blocks, and were identical in paired ambient and heated sub-plots. Dotted fill boxes represent the 8 internal plants used for end-of-season biomass harvests.

Figure S2

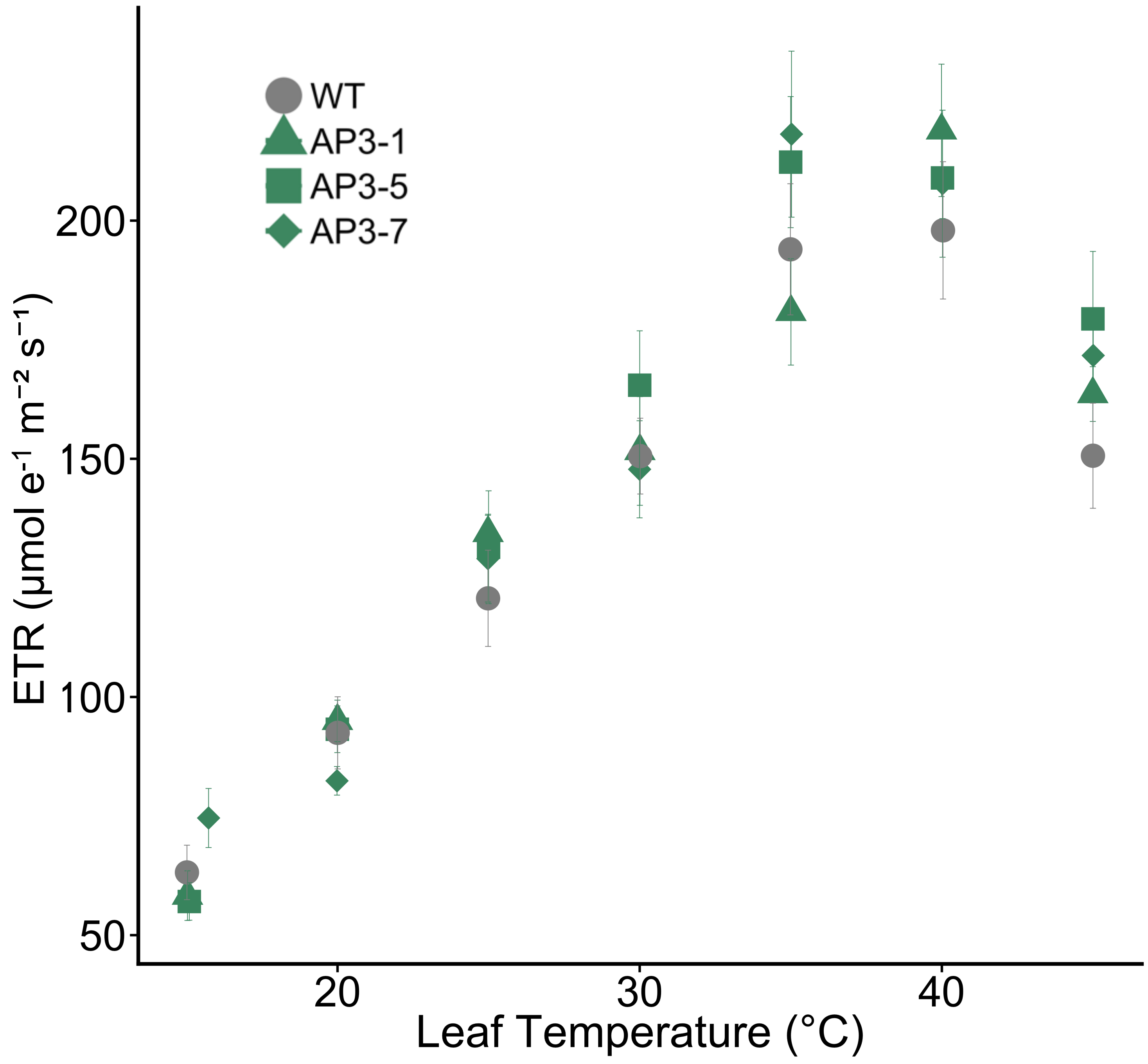

Supplemental Figure 2: Temperature responses of photosynthetic electron transport rate measured in greenhouse-grown AP3 and WT lines. Data are the mean and standard error for n=4-6 replicates. * indicates statistical differences at $P < 0.05$ between AP3 lines and WT based on repeated measures ANOVA and Dunnett's post-hoc comparison.

Figure S3

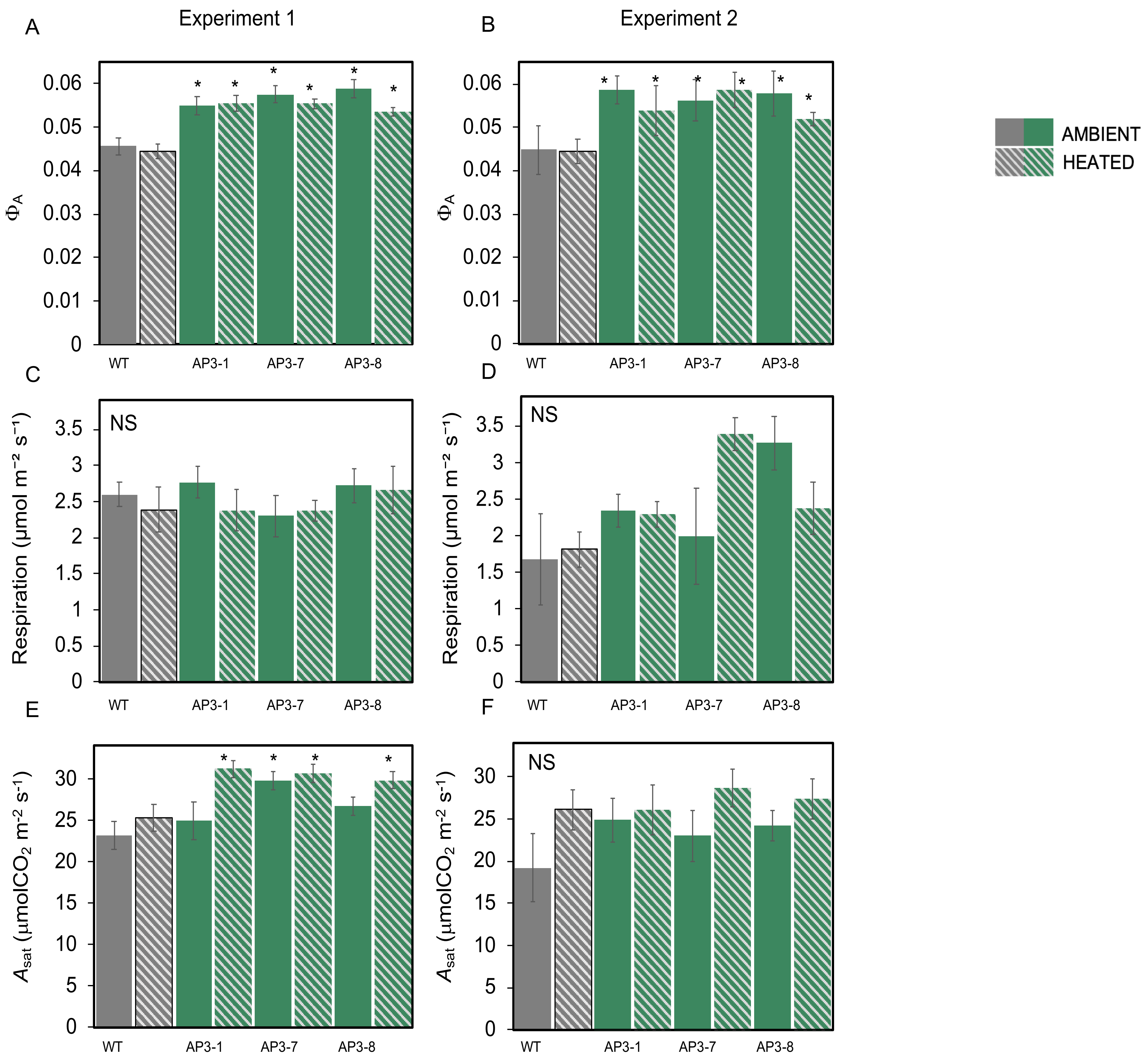

Supplemental Figure 3: Photosynthetic parameters estimated from light response curves. Apparent quantum efficiency of photosynthesis (Φ_A) (A,B), Dark Respiration (C,D) and light-saturated CO_2 Assimilation (E,F) of field-grown AP3 and WT lines in ambient and heated conditions. Φ_A was determined by linear regression of assimilation based on light-response curves for WT (gray) and AP3 (green) plants grown under ambient (solid) and heat (dashed) treatment. Data represent the mean and standard error for four (experiment 1) and three (experiment 2) plots. Letters represent statistical difference between each AP3 line and WT at $P < 0.1$ based on Dunnett's post-hoc comparisons following mixed effects model analysis.

Figure S4

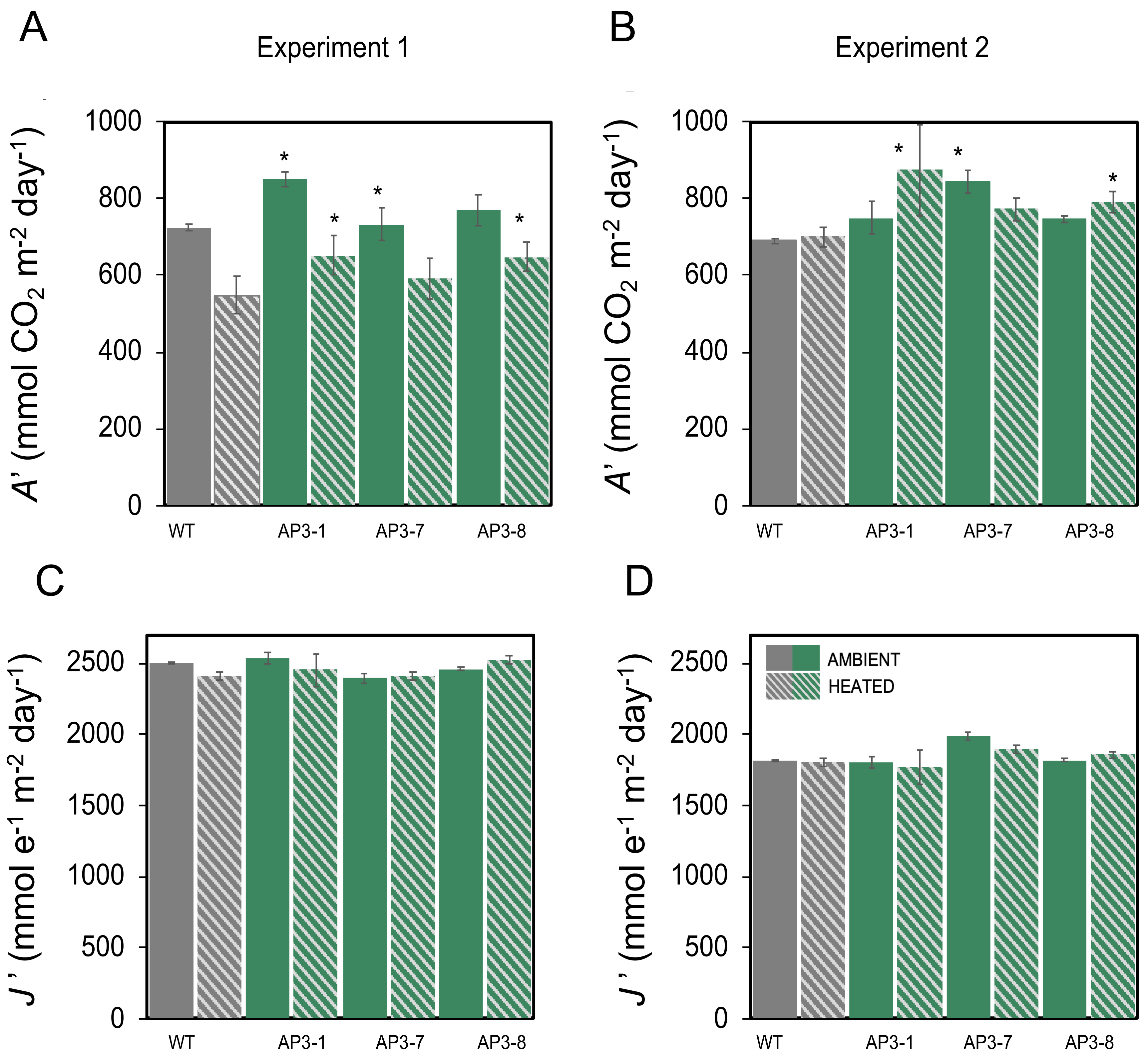

Supplemental Figure 4: Accumulated assimilation of CO_2 (A') in field-grown WT and three independent transformations of AP3 in ambient and heated conditions. A' was determined based on diurnal analysis of photosynthesis for WT (gray) and AP3 (green) plants grown under ambient (solid) and heat (dashed) treatment. Data represent the mean and standard error for four (experiment 1) and three (experiment 2) plots. Letters represent statistical difference between each AP3 line and WT at $P < 0.1$ based on Dunnett's post-hoc comparisons following mixed effects model analysis.

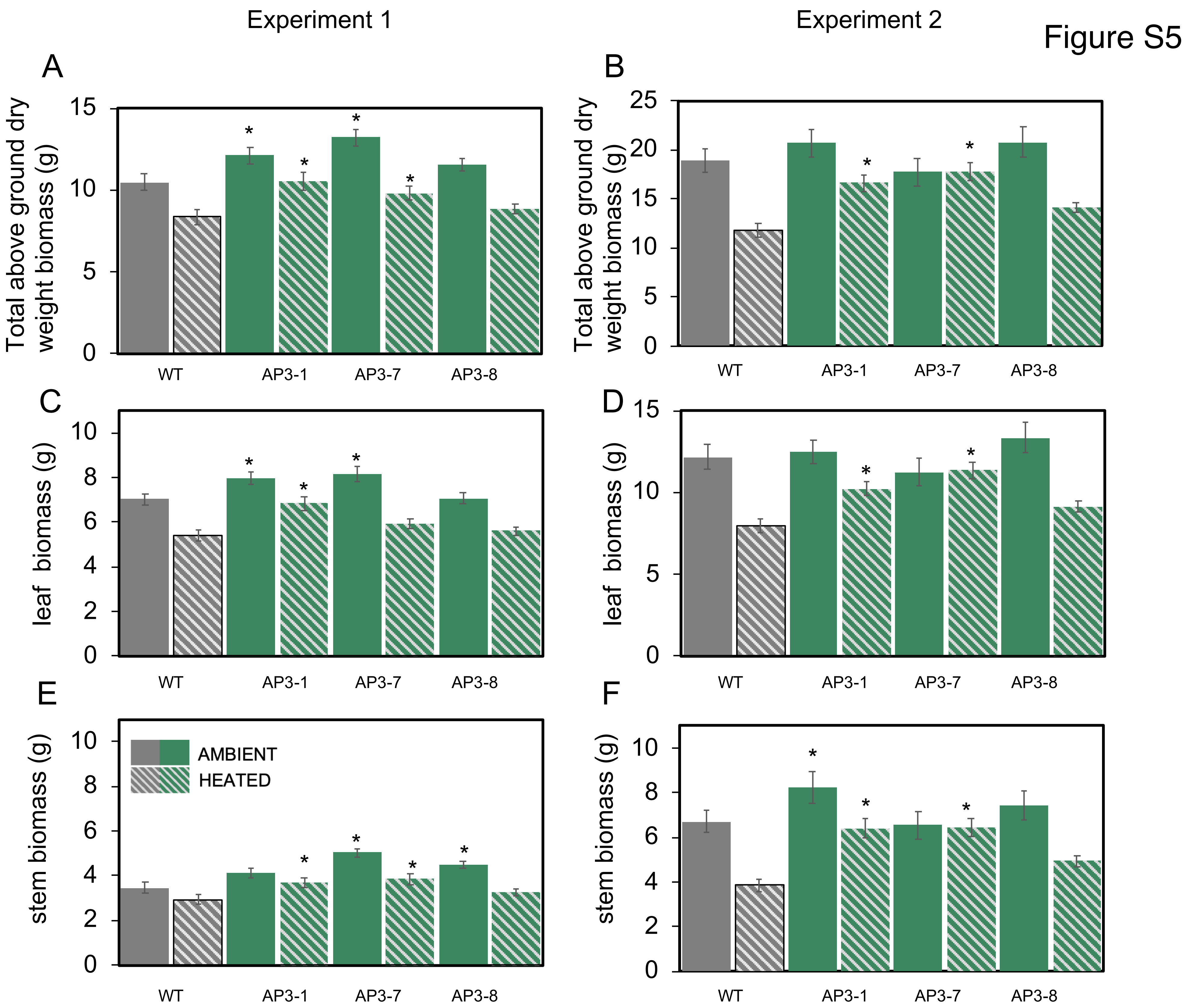

Supplemental Figure 5: Dry weight biomass of field-grown WT and 3 independent transformations of AP3 under ambient and heated conditions. Total (A, B), leaf (C, D), and stem (E, F) biomass of AP3 (green) and WT (gray) plants grown under ambient (solid) and heat (dashed) treatment from planting 1 (A,C,E) and 2 (B,D,F). Data represent the mean and standard error for four (experiment 1) and three (experiment 2) plots. Letters represent statistical difference at *P* < 0.1 based on Dunnett's post-hoc comparisons following mixed effects model analysis.

Figure S6

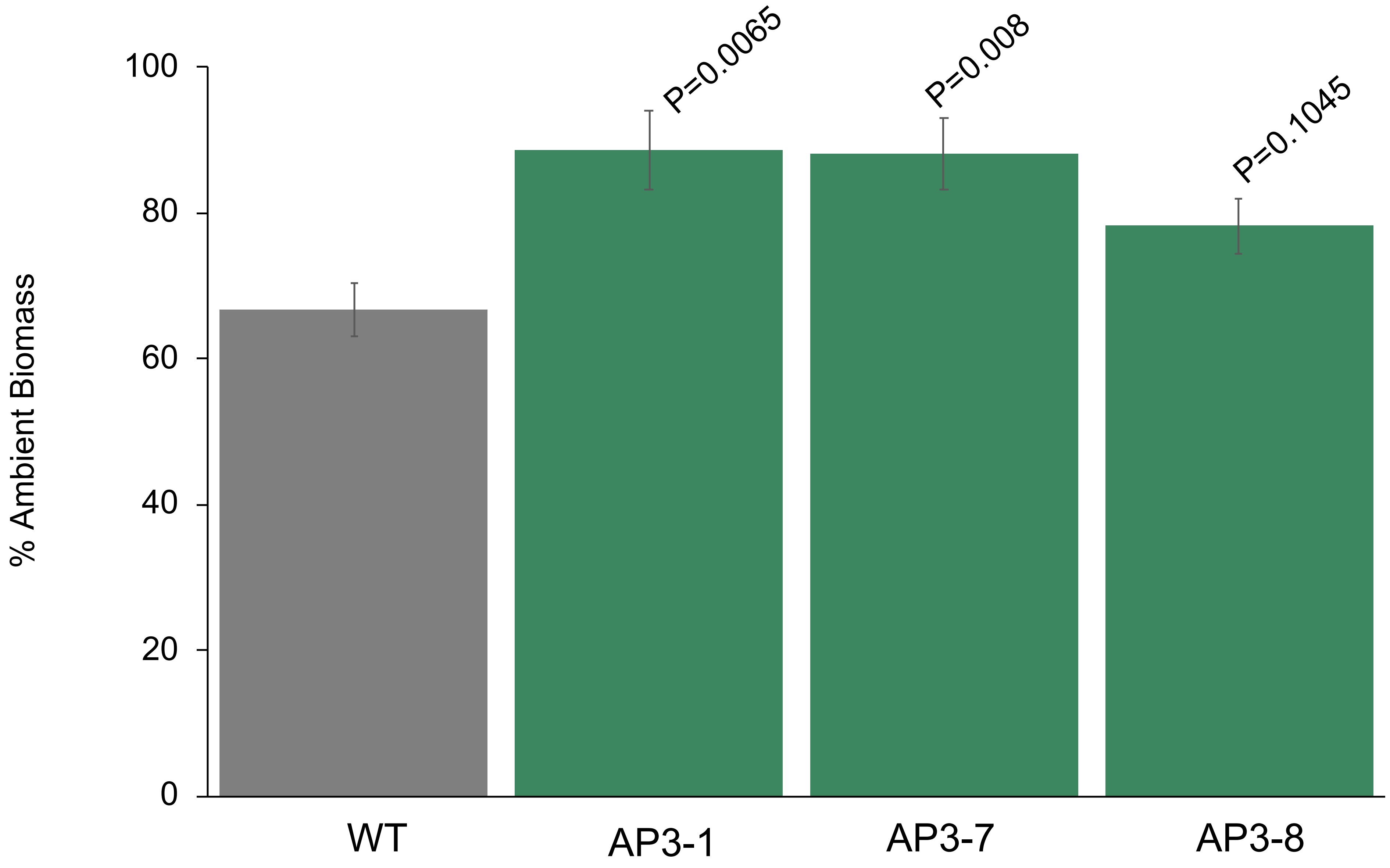

Supplemental Figure 6: Dry weight biomass retained under heating conditions relative to ambient conditions. Mean responses shown of WT (gray) and three independent transformations of AP3 (green) are the combined result of seven experimental plots over two planting experiments. Error bars represent standard error, and letters represent statistical difference at $P < 0.1$ based on Dunnett's post-hoc comparisons following mixed effects model analysis.

Table S1

Primer Name	Primer Type	Sequence
**L25 RT F**	QRT-PCR	‘CCCCTCACCACAGAGTCTGC’
**L25 RT R**	QRT-PCR	‘AAGGGTGTTGTTGTCCTCAATCTT’
**PLGG1 Nt RT-1F**	QRT-PCR	‘CTCAAATAAAGTTGAAATCCTTACAAAC’
**PLGG1 Nt RT-2R**	QRT-PCR	‘TCTTGGTAGGGATGAATTGGAC’
**RT-MS-001F**	QRT-PCR	‘GGGAATCTGAGTGGACATGTG’
**RT-MS-002R**	QRT-PCR	‘CCAGAATTGAGTGCGTTGATG’
**RT-GDH-001F**	QRT-PCR	‘AAGTTTATCCCCAAGGAGCG’
**RT-GDH-002R**	QRT-PCR	‘CACCTTCACTACCAGCTTCG’

Supplemental Table 1:
A list of primers used in this work

Table S2

		Value at 25°C	c	ΔHa
Bernacchi *et al.,* 2001		4.3		37.83
WT		4.6	15.6 ± 0.6[a]	34.8 ± 1.6[a]
AP3		4.6	13.3 ± 0.9[b]	29.2 ± 2.2[b]
	201-1	4.2	12.4 ±1.5[b]	27.2 ± 4[b]
	201-5	4.6	13.2 ± 1.6[b]	28.9 ± 3.6[b]
	201-7	4.3	14.2 ± 0.7[ab]	31.4 ± 1.8[ab]

Supplemental Table 2: The scaling constant (*c*) and heat of activation (ΔHa) of *Ci** measured in WT and AP3 plants. Parameters were calculated according to the equation Parameter = $\exp(c - \Delta Ha/RT_K)$, where R is the gas constant (8.314 $JK^{-1}mol^{-1}$), and T_K is the leaf temperature in Kelvin. Letters represent statistical differences at $P<0.05$ between AP3 lines and WT based on a one-way ANOVA and Tukey's post-hoc comparison.
